# Expression of EGFR and Molecules Downstream to PI3K/Akt, Raf-1-MEK-1-MAP (Erk1/2), and JAK (STAT3) Pathways in Invasive Lung Adenocarcinomas Resected at a Single Institution

**DOI:** 10.1155/2014/352925

**Published:** 2014-12-18

**Authors:** Alba Fabiola Torres, Cleto Nogueira, Juliana Magalhaes, Igor Santos Costa, Alessa Aragao, Antero Gomes Neto, Filadelfia Martins, Fabio Tavora

**Affiliations:** ^1^Department of Investigative Pathology, Argos Laboratories, 60175-047 Fortaleza, CE, Brazil; ^2^Department of Pathology, Messejana Heart and Lung Hospital, Rua Frei Cirilo 4290, 60846-190 Fortaleza, CE, Brazil; ^3^Department of Thoracic Surgery, Messejana Heart and Lung Hospital, 60846-190 Fortaleza, CE, Brazil; ^4^Department of Pulmonology, Messejana Heart and Lung Hospital, 60846-190 Fortaleza, CE, Brazil

## Abstract

Therapies targeting EGFR are effective in treating tumors that harbor molecular alterations; however, there is heterogeneity in long-term response to these therapies. We retrospectively analyzed protein expression of EGFR, Stat3, phospho-Akt, and phospho-Erk1/2 by immunohistochemistry in a series of resected cases from a single institution, correlated with clinicopathological variables. There were 96 patients, with the majority of cases being of low stage tumors (17 pT1a, 23 pT1b, 30 pT2a, and 18 pT2b). Histologic subtypes were 45 acinar predominant, 2 cribriform, 25 solid, 7 papillary, 11 lepidic, and 4 mucinous tumors. The EGFR score was higher in tumors with vascular invasion (*P* = 0.013), in solid and cribriform acinar histology, and in high stage tumors (*P* = 0.006 and *P* = 0.01). EGFR was more likely overexpressed in solid compared to lepidic tumors (*P* = 0.02). Acinar tumors had the highest rate of ERK1/2 positivity (19%). There was a strong correlation among positivity for ERCC1 and other markers, including STAT3 (*P* = 0.003), Akt (*P* = 0.02), and ERK1/ERK2 (*P* = 0.0005). Expression of molecules downstream to EGFR varied from 12% to 31% of tumors; however, the expression did not directly correlate to EGFR expression, which may suggest activation of the cascades through different pathways. The correlation of protein expression and the new lung adenocarcinoma classification may help in the understanding of activated pathways of each tumor type, which may act in the oncogenesis and drug resistance of these tumors.

## 1. Introduction

Lung cancer is the leading cause of cancer death worldwide. Most lung patients present with advanced stage disease and are not candidates for curative surgery; however, gene-targeted therapies offer hope for prolonged survival in advanced disease [1, 2]. A contemporary revolution in the treatment of nonsmall cell lung cancer (NSCLC) centers on biomarkers that predict who are more likely to respond to specific targeted therapies [3, 4]. These biomarkers are typically assessed by protein overexpression evaluated by immunohistochemistry, gene copy number evaluated by in situ hybridization, or specific mutations studied by a variety of PCR methods. These studies have been used mainly as predictors of response to the EGFR tyrosine kinase inhibitors (TKIs) [5, 6].

Until now, the major focus has been identification of EGFR mutated adenocarcinomas that respond to TKI. While therapies targeting EGFR and, more recently, ALK are effective in treating tumors that harbor molecular alterations, there is heterogeneity in results due to the complexity of signaling pathways involved both in oncogenesis and in acquired resistance. Therefore, analyses of other molecular signatures and profiles in protein expression may be critical in understanding the development in effective new drugs and combinations [7–11].

Activation of the EGFR results in activation of downstream signaling pathways, including the Ras-Raf-MKK extracellular signal regulated kinase (ERK) and lipid kinase phosphatidylinositol 3-kinase/Akt pathways [8, 12]. The activation of the downstream molecules Akt and the signal transduction and activator of transcription (STAT), which promotes cell survival, may be responsible for resistance to apoptosis induced by therapies [13]. Another pathway that may act in the inhibition of apoptosis is the ERK1/2 pathway [11, 14, 15]. Furthermore, enzyme repair cross-complementation group 1 (ERCC1) also plays a key role in excision repair enzyme pathways and its expression has been suggested to correlate with survival in lung carcinomas, as well as predicting sensitivity to platinum-based chemotherapy [16–19].

Recently, the International Association for the Study of Lung Cancer (IASLC), the American Thoracic Society (ATS), and the European Respiratory Society (ERS) have proposed a new subclassification of lung adenocarcinomas [20].

Herein, we studied a series of resected lung adenocarcinomas from a single institution reclassifying them with the new proposed ERS/ATS Classification [21, 22]. Then, we correlated the histologic subtypes with the expression of EGFR, Stat3, phospho-Akt, and phospho-Erk1/2, which are downstream signaling molecules that mediate the biological actions, and investigated the expression with clinicopathological variables. The main objectives of the study were to (1) evaluate the types of adenocarcinomas in a contemporary series using the new proposed classification, (2) evaluate expression of proteins related to the EGF oncogenic pathways, and (3) study the expression of these proteins in relation to established clinical and pathological characteristics.

## 2. Material and Methods

We conducted a retrospective review of pulmonary adenocarcinomas that were resected between 2009 and 2013 at the Messejana Heart and Lung Hospital, a reference center for lung cancer from Northeastern Brazil. All resected specimens were formalin fixed and stained with hematoxylin and eosin in the routine manner. The average number of slides from each case we reviewed in our study was 6.7 (range: 1–26).

Cases were reviewed by two independent pathologists with experience in pulmonary pathology and were classified according to the recent proposed classification of adenocarcinomas by the International Association for the Study of Lung Cancer/American Thoracic Society/European Respiratory Society [22–24]. The morphologic patterns, predominant histologic subtypes (lepidic, acinar, papillary, micropapillary, cribriform, and solid), presence of lymphovascular invasion, pleural invasion, and other clinical data were recorded. Percentage of solid and lepidic components was assessed semiquantitatively at 10% increments and recorded for each tumor. Tumors were divided into two groups, one of high-grade histology (predominant solid and cribriform tumors) and others. Sarcomatoid carcinomas, adenosquamous carcinomas, and preinvasive or minimally invasive lesions according to the aforementioned classification (atypical adenomatous hyperplasia, adenocarcinoma in situ, and minimally invasive adenocarcinoma) were not included in the study. High-grade histology (predominant solid or acinar with prominent cribriform gland formation) was compared with the other tumors and variables were compared [25, 26].

Clinicopathological characteristics including final pathologic stage, sex, and age were also recorded. In 22 of 96 cases, information about EGFR mutation status was available, and, in 39 of 96 cases, information on KRAS gene status was available and, while not being the main objective of this study, this data was also recorded.

Immunohistochemical studies were performed in one paraffin block chosen with a large percentage of tumor in every case. Antibodies for biomarkers related to EGFR, Raf-1-MEK-1-MAP, and JAK proteins were commercially obtained. These included* EGFR* (1 : 100, clone 31G7, Invitrogen, USA),* STAT3* (1 : 400, polyclonal, Spring Bioscience, USA), phospho-Akt (1 : 100, 98H9L8, Invitrogen, USA), phospho-ERK1/2 (1 : 100, polyclonal, Invitrogen, USA), and ERCC1 (1 : 100, clone 8F1, DBS, USA). Dilutions were determined by standard titration methods for optimal staining (strongest signal with least background staining). Methods followed standard procedures. Briefly, sections were deparaffinized in xylene, hydrated in graded ethanol, rinsed in distilled water, and placed in Citrate buffer, 10 mM, pH 6.0 with steam for antigen retrieval. The immunostaining was done with Novolink Max Polymer Detection System (Leica Biosystems, UK) as per manufacturer's recommendation.

Evaluation of the immunohistochemical studies was done using by semiquantitative analyses for EGFR results using the H-score previously described. In summary, this method assigns a continuous score of 0–300, based on the percentage of positive cells multiplied by the staining intensity (0, 1, 2, and 3) according to different staining intensity visualized at different magnifications [27]. Evaluation of the remainder of the antibodies considered a case positive if at least 10% of the tumor had intense positivity in the cell site characteristic of each antibody. A case was considered positive only if the stain was present in the predominant growth pattern. STAT3 expression is granular cytoplasmic; ERK1/2 and Akt are both nuclear (more specific) and cytoplasmic, while ERCC1 is nuclear.

Comparisons of means were performed using Student's *t*-test and of multiple categories were performed using ANOVA means table with Fisher's post hoc testing. Nonparametric comparison was performed using Mann-Whitney comparison means testing. Statistical analysis was performed using SAS software (Cary, NC).

## 3. Results

### 3.1. Clinical Data

There were 96 patients in the study, including 41 men (mean age 65 ± 10) and 55 women (62 ± 12). All “tumors were from” were resected specimens: 91 cases were lobectomies (3 bilobectomies) and 5 pneumonectomies; 55 from the right side, 34 from the left. All were adenocarcinomas diagnosed in “resections”. The majority of cases were low stage tumors (17 pT1a, 23 pT1b, 30 pT2a, and 18 pT2b) with only 8 cases with the final pathological stage pT3 and none pT4. There were 15 patients with pN1 disease and 1 patient with pN2 with no correlation between nodal status and pT stage. Available in 62 of 96 cases, information about smoking history elicited a positive history in 71% of cases, with no difference in age, pathological stage, and histologic type between smokers and nonsmokers.

The clinicopathological data are summarized on Table 1.

### 3.2. Histology

**Figure 1:**
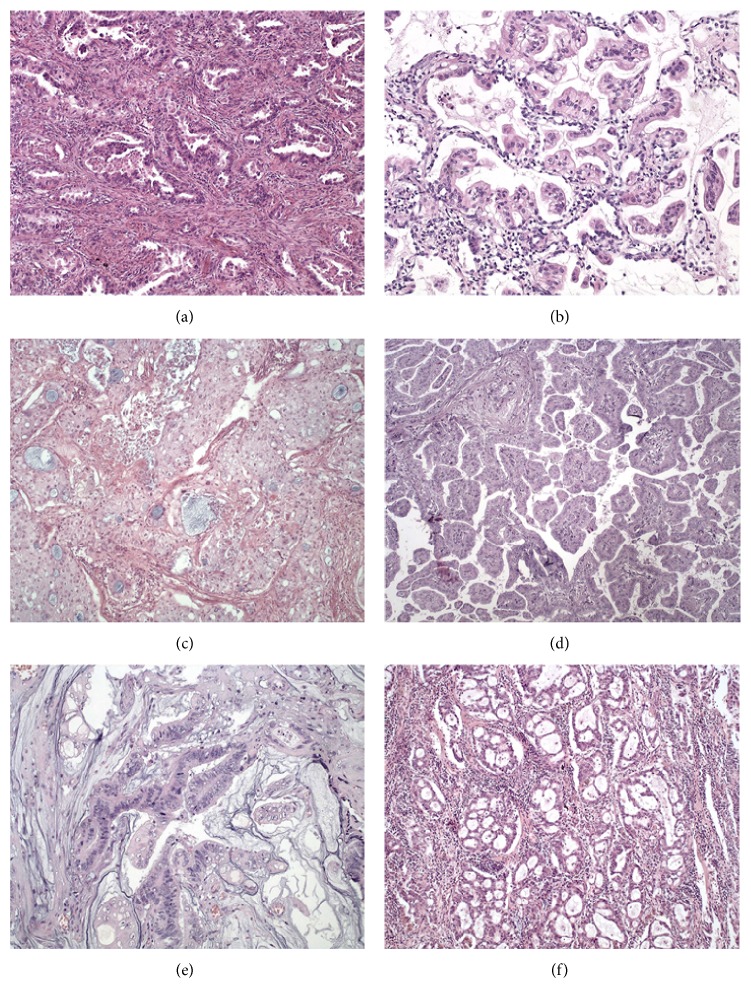
Morphologic features of lung adenocarcinoma according to the predominant growth pattern: (a) acinar; (b) lepidic; (c) solid; (d) papillary; (e) mucinous adenocarcinoma; (f) acinar tumor with prominent cribriform formation. All figures hematoxylin-eosin.

There were 58 well-differentiated adenocarcinomas, 26 moderately differentiated adenocarcinomas, and 11 poorly differentiated adenocarcinomas. Histologic subtypes encompassed 45 acinar predominant adenocarcinomas, 2 acinar tumors with prominent cribriform growth pattern, 25 solid predominant adenocarcinomas, 7 papillary adenocarcinomas, 11 lepidic predominant adenocarcinomas, and 4 mucinous/colloid tumors (Figure 1). Seventy-six cases (79%) had multiple patterns, and 20 were of the pure type. No tumor was predominantly micropapillary. Two cases were predominantly cribriform and were considered to be of high-grade histology along with predominantly solid tumors.

Thirty-five cases (38.3%) had ≥10% lepidic component, generally in the periphery of the mass (Figure 1) and 43 (44%) had ≥10% solid component. The percentage of solid component correlated with advanced T stage (*P* = 0.2) and did not correlate with age or sex. Both solid and papillary tumors were more likely to harbor lymphovascular invasion (*P* = 0.04), and predominant solid tumors also correlated with the presence of pleural invasion, independently of tumor size on multivariate analyses (*P* = 0.005). High-grade tumor histology correlated with pleural invasion (*P* = 0.02), vascular invasion (*P* = 0.01), and tumor size (*P* = 0.01) but did not correlate with nodal metastasis (pN stage) or age.

### 3.3. Immunohistochemistry

There were 46 cases positive for TTF-1, most often present in acinar predominant tumors (*P* = 0.05). No mucinous tumors were positive for TTF-1.

EGFR histologic score was performed in all tumors, 64 being negative and 32 being positive (cutoff above score of 200). Forty-six cases (48%) had a score of 0. Fourteen cases (15%) had a score of 300. The EGFR score was higher in tumors with vascular invasion (*P* = 0.013) and there was a trend towards tumors with pleural invasion (*P* = 0.06). High-grade histology and higher stage tumors (pT2 or pT3) also had significantly higher EGFR score (*P* = 0.006 and *P* = 0.01 resp.), and there was no correlation with age and sex. Across histologic subtypes, EGFR was more likely overexpressed in solid compared to lepidic tumors (*P* = 0.02).

**Figure 2:**
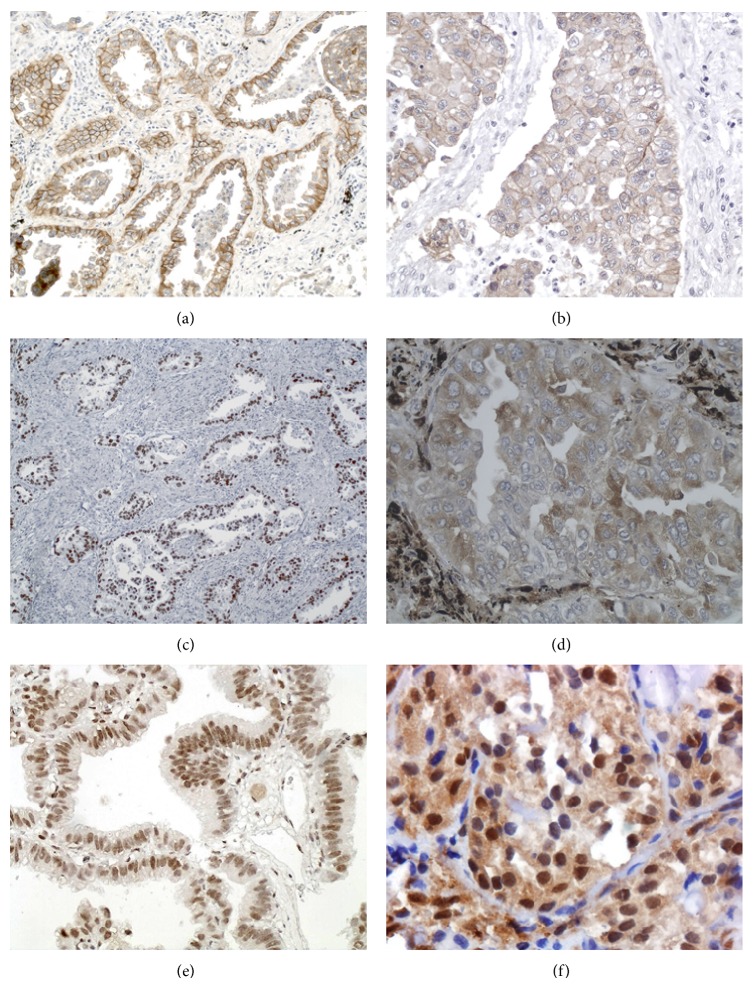
Immunohistochemical findings of different markers in lung adenocarcinomas. (a) EGFR positive acinar predominant tumor showing both distinct membranous and cytoplasm expression. (b) High power of EGFR positive solid predominant adenocarcinoma. (c) TTF-1 positive acinar tumor. (d) High power of STAT3 positive tumor with intense cytoplasm positivity. (e) ERCC1 positive lepidic predominant tumor, with intense nuclear positivity. (f) ERK1/2 expression in solid tumor showing both nuclear and cytoplasm reactivity.

**Figure 3:**
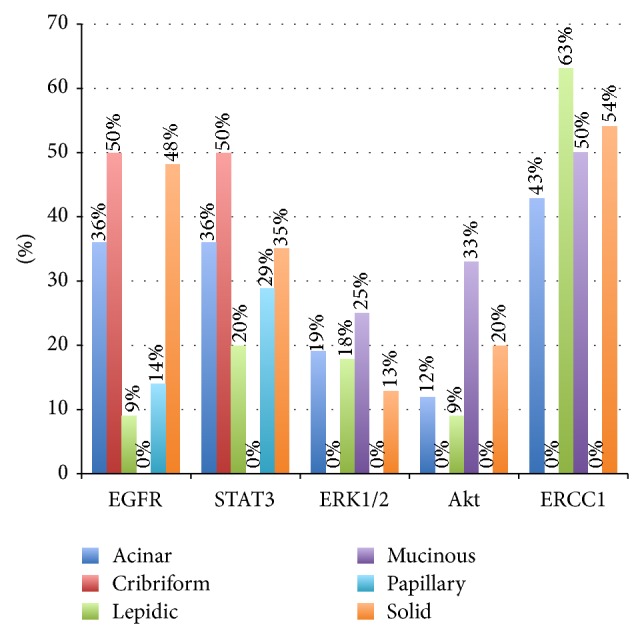
Percentage of expression of predominant histologic subtype of each immunohistochemical protein (EGFR, ERK1/2, STAT3, Akt, and ERCC1). The EGFR cutoff for positivity was the histologic score of 200.

The STAT3, ERK1/2, and Akt data are summarized in Table 2. STAT3 was positive in 29 tumors (32%), including 16 acinar, 1 cribriform, 2 lepidic, 2 papillary, and 8 solid type. ERK1/2 was positive in 14 tumors (15.5%), including 8 acinar, 2 lepidic, 1 mucinous, and 3 solid type. Akt was positive in 12 tumors, which included 5 acinar, 1 lepidic, 1 mucinous, and 5 solid. The results are summarized in Figures 2 and 3.

There was no correlation among protein expression of STAT3, Akt, and ERK1/2 with histologic subtype, clinical characteristics, or tumor stage. Acinar tumors had the highest rate of ERK1/2 positivity (19%). High-grade histology did not correlate with any of the aforementioned epitopes.

ERCC1 positivity was found in 38 cases (39%) and tumors were more likely lepidic or solid. No papillary tumor was ERCC1 positive. There was a strong correlation among positivity for ERCC1 and other markers, including STAT3 (*P* = 0.003), Akt (*P* = 0.02), and ERK1/ERK2 (*P* = 0.0005). Positive cases tended to present a lower EGFR score.

High expression of EGFR (score above 200) did not correlate with positivity of AKT, ERK1/2, or STAT3 (*P* > 0.5 for all proteins). Cases that were positive for STAT3 had a mean score of 123.4, while negative cases had a mean score of 99.1, with the correlation not being statistically significant (*P* = 0.3). Cases that were positive for Akt and ERK1/2 had lower EGFR score, with no statistical significance (*P* > 0.5).

## 4. Discussion

Lung adenocarcinomas are the most common histologic subtype and are part of the larger group of nonsmall cell lung cancer (NSCLC) that has been target of a recent evolution on the chemotherapeutical regimens based on molecular profiling. While NSCLC patients may harbor EGFR mutations in about 20% of the cases [28–30] and these tumors may respond well to EGFR tyrosine kinase inhibition, the responses are usually incomplete [31]. This phenomenon of drug resistance or failure may be explained in part by the activation of different oncogenic pathways or de novo mutations that activate downstream molecules and increase signal transduction. In this scenario, the identification of overexpressed molecules in different subtypes of lung cancer may help identify potential targets for combined therapy. For example, combining AKT inhibition with EGFR TKI may improve response in lung cancer patients harboring EGFR mutations [32].

We set out to determine the expression of molecules related or downstream to the EGFR receptor oncogenic development in lung adenocarcinoma subtypes according to the IASLC/ATS/ERS classification. The prognostic value of this classification by itself has been validated in retrospective studies, which include a previous study from our group [22, 33, 34]. To the best of our knowledge, the association between these epitopes and the new morphologic subtypes has not been hitherto investigated in this population. In addition, there has not been a contemporary study on lung cancer from South America and particularly Brazil, with emphasis on the new morphological classification and its relation with immunohistochemical expression outline. Protein expression is aligned directly with cellular activity and the proteomic profiling of tumors may help in the understanding of tumor progression, resistance mechanisms, and metastasis [35].

In the current series, the majority of tumors were of acinar subtype, followed by solid and lepidic variants, similar to other contemporaneous studies with the new classification [22, 33, 36]. Some other studies have tried stratifying invasive adenocarcinoma subtypes in architectural categories related to the aggressiveness of the tumors and predictive behaviour. Solid, micropapillary, and acinar tumors with prominent cribriform features are considered high grade, acinar and papillary are considered to be of intermediate grade, and predominantly lepidic tumors are considered to be of low-grade behaviour [26, 34, 37, 38]. Herein, we divided the tumors into high grade (solid predominant and cribriform) and others, to evaluate differences in expression between these two groups.

The main purpose of the study was to assess EGFR expression by immunohistochemistry and correlate with the morphologic subtypes according to the new adenocarcinoma classification. While currently only patients with tumors harboring identified mutations in the tyrosine kinase regions of the EGFR gene are candidates for targeted drugs, there is a body of literature enforcing the importance of EGFR expression in these tumors. Particularly, high EGFR expression may predict survival benefit from drugs such as cetuximab in patients with advanced NSCLC [27]. Interestingly, our data shows that, in this population, the EFGR mean score, assessed using the H-score method previously reported, was significantly higher in tumors with vascular invasion and higher T stage and in tumors with high-grade histology (mainly solid subtype). Li et al. have demonstrated similar correlation of aggressiveness with nodal status and overall status [12]. This finding is interesting in light of a few studies that have found that activating EGFR mutations is more common in tumors with acinar or lepidic subtype [39–41]. These studies have not, however, addressed EGFR expression by immunohistochemistry. The current study is in agreement with a study by Sholl et al. who have demonstrated both greater expression and EGFR gene amplification in solid tumors compared to other subtypes [42].

Studies of excision repair cross-complementing gene 1 (ERCC1) expression have shown correlation with prognosis and response to treatment gastric, pancreatic, cervical, and lung adenocarcinomas [10, 17, 43–46]. Particularly in lung adenocarcinomas, ERCC1 has been shown to be associated with unchanged EGFR mutation status [16], to predict response to cisplatin-based therapies and also to be of prognosis value by itself [19, 47, 48]. We have shown that there was no difference in ERCC1 expression among various morphological subtypes and also no statistical difference in the clinicopathological variables such as age, sex, tumor size, vascular invasion, pleural invasion, and high-grade histology, suggesting that its expression may be an independent prognostic variable, similar to other studies [49, 50].

We also analyzed the expression of the PI3K/AKT pathway by the expression of the phosphorylated AKT and also the mitogen-activated protein kinase/extracellular signal regulated kinase (MAPK/ERK) pathway by the expression of the phosphorylated ERK1/ERK2 proteins. Activation of AKT has been associated with disease progression in tumors with wild-type EGFR, suggesting that it plays a role in contributing to gefitinib resistance [9]. Other studies have shown that ERK1/ERK2 expression is linked with increase in cell proliferation and also correlates with advanced stage and lymph node metastases [14, 15]. In our tumors, AKT positivity correlated inversely with vascular invasion. This information may be important especially in small samples, given that most patients present with unresectable tumors and no other tissue may be available for protein or DNA analyses. Although this series has a selection bias on resected specimens, analyzing a large tumor volume is important in identifying these markers that are of prognostic value. Although EGFR activation may play a role in both ERK and AKT pathways stimulation, we have found no correlation between EGFR mean score and expression of activated ERK [51, 52]. This may suggest that there are different oncogenic pathways in tumors with high EGFR expression profiles, and this information may help understand resistance mechanisms.

Signal transducer and activator of transcription (STAT) 3 is involved in cell proliferation and apoptosis. In lung adenocarcinomas, STAT3 is one of the most important signaling mediators in both normal and EGFR-mutated tumors [53–55]. It is persistently activated in about 50% of NSCLC tumors and shows correlation with EGFR mutation status [56]. In one study, using lung carcinoma cell line, EGFR inhibition did not block STAT3 phosphorylation and growth arrest, suggesting that upstream pathways, such as JAK and IL6 receptor, may play a role in activating STAT3 and contributing to oncogenesis in lung tumors, even with EGFR activation [53]. In this series, expression of phosphorylated STAT3 was found in all subtypes, except the mucinous variant, but there was no correlation with clinicopathological variables or EGFR mean expression. It is remarkable that mucinous tumors also did not show TTF-1 expression. It has been postulated that the JAK-STAT signaling pathway may be involved in the oxidative stress-induced decrease in expression of surfactant protein genes, such as TTF-1, which may play a role in oncogenic of this specific morphologic subtype [57].

The current study has a few weaknesses. The main one is the lack of mutational analyses. Since it has been known that specific mutations in the 18–21 exons of the EGFR gene predict response to targeted therapies, it would be of great clinical value to compare the expression of each of the studied proteins with genetic information.

The second weakness refers to lack of follow-up information. Given the recent retrospective review, information regarding follow-up status is lacking. It would be of great interest to also evaluate the expression of the aforementioned markers with information regarding tumor progression of relapse.

We also acknowledge a selection bias in including only resected tumors, but, to better evaluate the extent of protein expression and morphologic subtypes, we chose not to include unresectable tumors with the diagnoses made on small biopsies or cytology specimens.

We have shown a series of resected cases of adenocarcinomas from a single institution and estimated the expression of proteins that may be target for current or future drug development. In this particular population of low stage, resectable lung tumors, information regarding tumor epitope expression may greatly contribute to the understanding of disease recurrence and/or responsiveness to adjuvant therapy.

## Figures and Tables

**Table 1:** Clinicopathological characteristics of cases.

	Number of patients (%)
Sex	
Male	41 (57)
Female	55 (42)
Age	
<60 yo	45
>60 yo	50
Pathological T stage	
pT1a	17 (18)
pT1b	23 (24)
pT2a	30 (31)
pT2b	18 (19)
pT3	8 (8)
Pathological N stage	
N0	80 (80)
N1	15 (16)
N2	1 (1)
Tumor differentiation	
Well differentiated	58 (61)
Moderately differentiated	26 (27)
Poorly differentiated	11 (11)
Histologic subtypes	
Acinar	45 (46)
Acinar cribriform	2 (2)
Solid	25 (26)
Papillary	7 (7)
Lepidic	11 (11)
Mucinous	4 (4)

**Table 2:** Expression of EGFR, p-ERK1/2, p-STAT3, ERCC1, and Akt by immunohistochemistry, and clinicopathological characteristics in 96 patients with lung adenocarcinoma.

Group	EGFR mean score	*P* value	pERK1/2 positivity (%)	*P* value	p-STAT3 positivity (%)	*P* value	p-Akt positivity (%)	*P* value	ERCC1 positivity (%)	*P* value
Sex										
Male	122.4	0.19	7 (7.61)	0.5	14 (15.3)	0.2	4 (4.3)	0.4	18 (20.4)	0.6
Female	91.0		7 (7.61)		15 (16.4)		8 (8.6)		21 (23.8)	
Age										
Below 60 years	82.2	0.14	3 (3.2)	0.17	8 (8.7)	0.2	5 (5.3)	0.8	12 (13.6)	0.17
Above 60 years	177.8		11 (11.9)		21 (23)		7 (7.5)		27 (30.6)	
Size/stage										
T1	70.0	**0.01**	8 (8.7)	0.22	11 (12)	0.7	6 (6.4)	0.5	18 (20.4)	0.4
Greater than T1	129.1		6 (6.5)		18 (19.7)		6 (6.4)		21 (23.8)	
Vascular invasion										
Yes	80.1	**0.01**	2 (2.2)	0.05	11 (12.3)	0.9	2 (2.2)	**0.05**	12 (13.9)	0.2
No	141.7		12 (13.3)		18 (20.2)		11 (12)		26 (30.2)	
Pleural invasion										
Yes	90.4	0.07	2 (2.2)	0.3	8 (8.9)	0.5	1 (1.1)	0.1	10 (11.6)	0.7
No	142.1		13 (13.9)		21 (23.6)		11 (12)		28 (32.5)	
High grade histology										
Yes	155.5	**<0.01**	3 (3.2)	0.5	9 (9.8)	0.6	5 (5.3)	0.3	13 (14.7)	0.5
No^*^	84.4		11 (11.9)		20 (21.9)		7 (7.5)		26 (29.5)	

^*^Predominantly solid or acinar cribriform tumors (high grade) versus other subtypes.
